# Draft Genome Sequence of *Saccharomyces cerevisiae* Barra Grande (BG-1), a Brazilian Industrial Bioethanol-Producing Strain

**DOI:** 10.1128/genomeA.00111-17

**Published:** 2017-03-30

**Authors:** Natalia Coutouné, Aline Tieppo Nogueira Mulato, Diego Mauricio Riaño-Pachón, Juliana Velasco de Castro Oliveira

**Affiliations:** Brazil Laboratório Nacional de Ciência e Tecnologia do Bioetanol (CTBE), Centro Nacional de Pesquisa em Energia e Materiais (CNPEM), São Paulo, Brazil

## Abstract

Here, we present the draft genome sequence of *Saccharomyces cerevisiae* BG-1, a Brazilian industrial strain widely used for bioethanol production from sugarcane. The 11.7-Mb genome sequence consists of 216 scaffolds and harbors 5,607 predicted protein-coding genes.

## GENOME ANNOUNCEMENT

The Brazilian process of bioethanol production is characterized by industrial fermentations in large tanks (0.5 to 3 million liters) and high yeast cell densities (10 to 15% wt/vol). A complete fermentation cycle is performed within a very short period (6 to 12 h), achieving ethanol concentrations of 7 to 11% (vol/vol). In addition, after a treatment with sulfuric acid (pH 1.8 to 2.5) the yeast cells are returned to tanks to start a new fermentation batch (1). Accordingly, Brazilian industrial strains must be able to deal with several stressing agents, and the genomes of the strains currently published (2, 3) show significant adaptive genetic variability (4).

*Saccharomyces cerevisiae* strain Barra Grande (BG-1) is a heterothallic strain, isolated from a Brazilian distillery in 1989-1990 (4). BG-1 has tolerance to high temperature, does not flocculate, ferments with low foam production, and is adapted to the Brazilian fermentation process. Its genome was sequenced on the Illumina HiSeq 2500 system, using a paired-end library with an insert size of 300 bp, and three mate-pair libraries with insert sizes of 3 to 4 kb, 5 to 7 kb, and 8 to 11 kb, producing 4,187,211, 3,605,199, and 4,578,254 sequences, respectively. The reads were processed with the FastQC (5), NextClip (only the mate-pair reads) (6), and Trimmomatic (7) tools. Genome assembly was carried out with SPAdes version 3.7.1 (8), Pilon version 1.16 (9), and Redundans (10). Assessment of ploidy level was carried out with ploidyNGS (11). BG-1 is a diploid organism. The haploid nuclear genome is assembled in 215 scaffolds, and additional scaffold contains the full 2-µm plasmid sequence. The nuclear genome has a total length of 11,691,159 bp and an *N*_50_ of 337,041 bp, with a coverage of 234×. The 2-µm plasmid has a length of 5,623 bp, with a coverage of 12,679×. The average G+C content of the nuclear genome is 38.25%, which is similar to *S. cerevisiae* S288C (~38%) (12). Gene prediction and annotation was carried out with the Funannotate pipeline (13). A total of 5,607 putative protein-coding genes were identified, and 89.38% have clear homologs in *S. cerevisiae* S288C (above 98% similarity). We found 280 genes coding for tRNAs, and the rRNA operon is collapsed into a single scaffold. The BG-1 draft genome sequence provides a source of information for elucidating the genetic mechanisms underlying the fermentation process and stress tolerance.

### Accession number(s).

This whole-genome shotgun project has been deposited at DDBJ/EMBL/GenBank under the accession number MSHP00000000. The version described in this project is the first version, MSHP01000000.
